# Metal Exposures, Noise Exposures, and Audiometry from E-Waste Workers in Agbogbloshie, Ghana

**DOI:** 10.3390/ijerph18189639

**Published:** 2021-09-13

**Authors:** Krystin Carlson, Niladri Basu, Julius N. Fobil, Richard L. Neitzel

**Affiliations:** 1 Department of Environmental Health Sciences, University of Michigan, Ann Arbor, MI 48109-2029, USA; krystin@umich.edu; 2 Faculty of Agricultural and Environmental Sciences, McGill University, Montréal, QC H9X 3V9, Canada; niladri.basu@mcgill.ca; 3 Department of Biological, Environmental & Occupational Health Sciences, School of Public Health, University of Ghana, Accra P.O. Box LG13, Ghana; jfobil@ug.edu.gh

**Keywords:** mixtures, occupational health, noise, lead (Pb), noise notch, hearing loss, ototoxicity, e-waste, Ghana, noise induced hearing loss

## Abstract

Metals, such as lead, may be ototoxic, but this property is not well understood, especially in conjunction with noise. This cross-sectional study investigated hearing, noise, and metal biomarkers in informal electronic waste (e-waste) recycling workers in Accra, Ghana. Workers (N = 58) participated in audiometric testing, a survey, blood collection, and personal noise dosimetry. Sixty percent of participants displayed audiometric notches indicative of noise-induced hearing loss (NIHL). Most workers (86%) reported high noise while working. Daily average noise levels were in the range 74.4–90.0 dBA. Linear regression models indicated participants who lived at Agbogbloshie Market for longer periods were significantly associated with worse hearing thresholds at 4 and 6 kHz. The models did not identify blood levels of lead, mercury, or cadmium as significant predictors of worse hearing thresholds or larger noise notches, but increased levels of selenium were significantly associated with better hearing at 6 kHz. Models of thresholds at 4 and 6 kHz were improved by including an interaction term between the maximum noise exposure and the level of zinc in whole blood, suggesting that zinc may protect hearing at lower noise levels, but not at higher levels. Further study of the relationships between elements, noise, and NIHL is needed.

## 1. Introduction

Worldwide, 466 million people are estimated to suffer from hearing loss (HL) and global prevalence is increasing [1]. The prevalence of adult-onset HL in developing nations, a large portion of which is due to occupational noise [2], is also increasing [3]. HL has profound impacts on human health and quality of life as well as a wide variety of adverse social, psychological, occupational, and educational outcomes [4]. Individuals with HL experience detrimental impacts on educational achievements and employment status, as well as challenges within the workplace [5,6].

One type of HL, noise-induced HL (NIHL), is completely preventable but also unfortunately prevalent. In the US, one in four adults has indications of NIHL and about one in three adults working in noise have NIHL [7]. Estimating the exact prevalence of NIHL is challenging as many individuals in the US either do not have access to, or do not give priority to, their hearing health, even when they would benefit from treatment. Only three in ten US adults under age 35 with hearing impairment will obtain a hearing aid [8].

With such high prevalence of HL, understanding how chemicals in the environment as well as drugs taken therapeutically may be linked to HL is important. Pharmaceuticals such as aminoglycosides are well known to cause negative outcomes in the auditory system [9], however, the emerging ototoxic effects of metals such as lead (Pb), mercury (Hg), and cadmium (Cd) are less understood [10,11]. Metals occurring in occupational exposures are an emerging area of ototoxicity research [12,13]. The US National Institute for Occupational Safety and Health (NIOSH) has highlighted the need for further research examining the relationship of metals to HL, especially those occurring in mixtures [14].

Elevated exposures to Pb, Hg, and Cd occur in many occupational settings [15,16,17], and environmental contamination with these metals is common in industrial and post-industrial communities [18]. Though the precise mechanisms by which Pb, Hg, and Cd exposure produce ototoxicity are not yet known, these three metals have neurotoxic properties and can induce oxidative stress imbalances [19,20], which is a mode of action fitting with prevailing hypotheses of noise trauma and drug-induced HL.

A number of epidemiological studies indicate that exposures to metals, in particular Pb and Cd, may be associated with an increased risk of HL [21,22,23,24]. American adolescents with higher Pb and Cd exposures have shown a greater likelihood for HL [22]. In a more aged population, X-ray fluorescence has been used to measure life-time accumulated bone Pb levels, and levels of Pb in bone were significantly associated with threshold increases at higher frequencies [23]. Park et al. (2010) also found increases in HL with bone Pb levels, even after accounting for occupational noise exposures [23]. Occupational exposures to Hg have also been associated with HL [25,26] in humans and Hg has been associated with adult-onset HL in monkeys [27].

Metal mixtures can evoke a synergistic response [28], and synergism and potentiation interactions have been noted for known non-metal ototoxicants like styrene, aminoglycosides, and jet fuel [29,30,31]. At least one study has suggested a possible interaction between noise and Pb exposure [32]. A handful of studies have assessed the contributions of Pb and noise to HL [23,32,33,34], but only two have evaluated the effects of combined exposures to Cd and noise [35,36]. No studies have yet evaluated the potential interactions between noise and metal biomarkers alongside nutritional essential element levels. A better understanding of multi-factorial exposures is necessary to prevent or reduce the risk of HL among workers.

While Pb, Hg, and Cd exposures in high-income countries are relatively well-controlled, the same is not true in low- and middle-income countries. In Ghana and other West African countries, for example, the high demand for electronic and electrical equipment (EEE) at affordable prices has resulted in massive importation of mostly secondhand EEE from developed countries [37,38]. Though Ghana, as of 2003, is a signatory of the Basel Convention [39], which forbids transboundary movement of hazardous wastes, exporters send unusable EEE into Ghana every year under the pretense of donating second-hand EEE [37,40]. These waste electronics are commonly referred to as “e-waste.” In 2009, 35% of secondhand EEE imports to Ghana (a total of 280,000 tons) were non-functioning, illegal e-waste [40] and were typically recycled by informal workers. E-waste flows to Ghana were projected to at least double in 2020 [41].

The informal sector in Ghana largely processes e-waste in two ways: manual dismantling with rudimentary tools and open-air burning [41]. Many of the workers are young, and few use any personal protective equipment [41,42]. Reusable parts and resalable copper, aluminum, gold, silver, iron, and brass are extracted and sold from electronic and electric products such as televisions, monitors, computers, audio equipment, cameras, printers, telephones, mobile phones, and household appliances [41,43]. However, e-waste contains metals hazardous to human health, including Pb, Cd, and Hg, as well as plastic components, which when burned produce toxic organic compounds [38]. Several studies at Agbogbloshie Market, Ghana’s largest e-waste site for many years, have found elevated levels of Cd and Pb in the blood and urine of e-waste workers compared with reference sites [42,44,45] and elevated concentrations of Pb and Cd in dust at the site and its surroundings [46].

The aims of this study were three-fold. First, we characterized the blood element levels, occupational habits, and noise exposures experienced by e-waste workers at the Agbogbloshie Market. Second, we assessed the associations of both essential and toxic elements in whole blood from e-waste workers with pure-tone audiometric thresholds as well as measures of NIHL, or noise notches (NN). Third, we assessed potential interactions between levels of essential elements, levels of toxic elements, and noise.

## 2. Materials and Methods

Informed consent was obtained from all subjects involved in the study. The study protocols were approved by the Institutional Review Boards at the University of Ghana, the University of Michigan, and McGill University. In addition, endorsement to carry out research was sought from the local chief of the Agbogbloshie Scrap Workers who allowed our research team to enter the community to conduct the study.

### 2.1. Participant Recruitment

Recruitment has previously been published and described [44]. Participants were recruited from Agbogbloshie Market with assistance from the local Chairman of the Greater Accra Scrap Dealer’s Association (who nominally oversaw the site, though employed no workers directly), as well as from three translators, one of which was a tribal chief in Northern Ghana. The first eight participants were approached directly by the chief, then subsequent workers were recruited by previous participants; a small fraction were recruited directly by research staff. All participants were over the age of 18 and had worked at Agbogbloshie for at least the past six months. Often, workers were recruited in clusters of two to five people working on similar waste materials and in similar locations.

Participants were transported, at no cost, to the Ghana Post Clinic, near to Agbogbloshie. At the clinic, participants underwent five study procedures, described in detail below. All study procedures were facilitated by a translator. Participants were compensated (equivalent of one day’s wages) and transported at no cost back to Agbogbloshie Market.

### 2.2. Health Evaluation

A brief medical examination was conducted for each participant. Information recorded during the exam included: current health insurance, height, weight, waist circumference, hip circumference, numbers and sites of work-related scars or deformities, skin disorders, and any other health complaints.

An outdoor clinic at the e-waste work site was held at the conclusion of data collection to allow participants to meet with the doctor again and go over any health concerns discussed during the survey or not addressed within our study. Medications for common fungal infections and malaria were made available to those needing treatment.

### 2.3. Hearing Assessment

An audiometric test was administered using an OtoPod M2 portable audiometer (Otovation, Inc., Portland, OR, USA). We obtained hearing threshold levels (HTLs) at the frequencies 500, 1000, 2000, 3000, 4000, 6000, and 8000 Hz on both ears. Two thresholds on both the right and left side were collected at 1000 Hz for verification of data quality. Background noise from the surrounding environment was attenuated with ear cups and insert headphones, which were estimated to reduce noise exposures by approximately 40 dB across the frequency range of interest. Background noise levels were checked prior to the start of each audiometric test; in the event of high background noise levels (>50 dBA), the test was interrupted until the noise levels declined. All testing was conducted by a technician certified by the Council for Accreditation in Occupational Hearing Conservation (CAOHC).

### 2.4. Survey

A survey was administered to participants to assess various occupational- and hearing-related factors. Surveys were filled out for the participants by the translators who administered them. Some respondents did not know their exact age and so it was noted when this was estimated. Survey items included demographic information about sex, age, education, marriage, income, hours worked per day, smoking habits, and living arrangements. Other risk factors for HL (e.g., congenital HL, ear infections, etc.), along with self-reported hearing status, were assessed to control for possible confounding. The items specific to hearing status included: whether participants had problems with their hearing, and if so, when they started having these problems; whether a doctor had diagnosed any HL; and whether they were taking any medications for this diagnosis. Additional items inquired about participants’ perceived occupational and non-occupational noise exposure and annoyance associated with these exposures. Translators qualified “work in loud noise” as working in an environment where you need to raise your voice to talk to someone beside you. The questionnaire collected further information on occupational history and exposures, socioeconomic status, occupational and residential noise exposures, use of personal protective equipment, and dietary intake.

### 2.5. Blood Biomarkers

Concentrations of elements were measured in whole blood using methods detailed previously by Srigboh et al. (2016) [44]. Briefly, a trained phlebotomist collected a blood sample from each participant using standard laboratory procedures. Blood was drawn in a Vacutainer tube and refrigerated. Samples were analyzed at McGill University using inductively-coupled plasma-mass spectrometry (ICPMS, Varian 720, Agilent, Santa Clara, CA USA) for levels of Pb and Cd, and a direct mercury analyzer (DMA) for total mercury (Hg) levels. Besides these three primary elements of interest, through the ICPMS analysis we also measured a number of essential and non-essential elements: selenium (Se), manganese (Mn), copper (Cu), iron (Fe), zinc (Zn), and arsenic (As).

### 2.6. Personal Noise Measurements

Noise exposures were assessed over a nominal 24-h period on each subject. Measurements were collected using a personal dosimeter (Etymotic Research ER-200D) that datalogged the equivalent continuous average exposure (L_EQ_) and maximum (L_max_) noise levels every 3.75 min over the 24-h period; this datalogging interval was the only one available on the device. Dosimeters were set to a slow response time and calibrated to manufacturer specifications. The measurement range of the dosimeter was 70–130 dBA, and the dosimeter was configured according to the 24-h exposure guidelines of the World Health Organization [47], since noise exposure regulation is poorly enforced in Ghana. No measurement threshold was used. The monitored period was intended to include work and non-work activities such as prayers, exercise, and sleep. Activity times and durations were collected following the measurement period using a separate researcher-administered daily activity log to allow for determination of noise levels during specific activities, as well as over the entire 24-h period. L_EQ_ and L_max_ data were used in linear and logistic modeling for differing time periods of overall, occupational, and non-occupational. Occupational noise exposures were converted to 8-h time-weighted average (*TWA*) levels to allow for comparison to occupational exposure limits using Equation (1):

(1) $$TWA=10\times log_{10}\left[\;1/128\int_{i=1}^{N}1\times 10^{L_{AEQ}/10}\right]$$

where *L_AEQ_* is a 3.75-min average equivalent continuous noise level, *N* is the total number of 3.75-min intervals, *i*, in the measured shift, and 128 is the number of 3.75-min intervals in a 480-min (i.e., 8-h) period. Normalizing all exposures to an 8-h TWA allows for direct comparison of exposures of differing measurement durations.

### 2.7. Analysis

Statistical analyses were performed in R (version 3.5.1, R: A Language and environment for statistical computing. R Foundation for Statistical Computing, Vienna, Austria: www.R-project.org, accessed on 18 June 2017) using RStudio (version 1.1.456, RStudio PBC, www.rstudio.com/, Boston, MA, USA). Descriptive statistics were computed for all variables. Significance was determined by *p*-values below 0.05 and borderline significance was determined by *p*-values less than 0.10.

Both L_EQ_ and L_max_ levels were explored in bivariate and regression analyses. Numerous 3.75 min dosimeter intervals had values of 0 dBA; these indicated measured noise levels that were below the dosimeter’s threshold of 70 dBA for the entire 3.75 min interval. To account for this limit of detection issue, we replaced these 0 dBA values with a value of 70/(√2) or 50 dBA [48].

In addition to analysis of survey items on primary jobs and e-waste activity involvement, a variable to describe work activity diversity, WorkD, was created by dividing the number of different e-waste activities workers reported performing in the past three months by the total number of activities evaluated. This variable was intended to summarize the diversity of a worker’s job activities as a surrogate for the variability in their exposure to various elements in e-waste. A few workers were not asked about all activities on the survey; in these cases, the denominator used was the number of activities for which responses were recorded. One participant who did not report performing any of the activities had a WorkD = 0 and was removed from further analyses based on this statistic.

The outcome of interest, HTLs in dB HL, was explored in several ways. We analyzed hearing thresholds at each audiometric test frequency; for these analyses, larger positive numbers indicate a greater degree of HL. Since NIHL is characterized by the appearance of worse hearing thresholds at 4 and/or 6 kHz (the so-called 4 kHz noise notch, NN) [49], we also computed several NN indices to evaluate the presence and area of audiometric notches. These indices calculate NN as a negative number, with larger negative numbers indicating a worse notch. All NN calculations consisted of a background average threshold compared to a threshold at a frequency or average across multiple frequencies at the expected notch location(s). The notch criteria considered are shown below in Equations (2)–(5) [50]:

NN_A_ = (average of 1 and 8 kHz) − (average of 3, 4, and 6 kHz) (2)

NN_B_ = (average of 2 and 8 kHz) − (average of 3, 4, and 6 kHz) (3)

NN_C_ = (average of 3 and 8 kHz) − (average of 4 and 6 kHz) (4)

NN_D_ = (average of 3 and 8 kHz) − (6 kHz) (5)

Bivariate relationships between all variables (e.g., survey responses, work activities, measured noise exposures, blood element levels, and HTLs) were evaluated using non-parametric Spearman correlation coefficients and scatterplots. Variables showing moderate (>0.4) or greater correlations with HTLs or the various NN values were further explored in a series of univariate linear regressions. Due to the possibility of low frequency noise occurring during audiometry testing (especially 500 Hz), and potential subsequent threshold masking, we limited analysis only to higher frequencies.

Finally, a series of multivariate linear regression models was developed utilizing variables which showed significance and borderline significance to assess the effects of various predictors identified *a priori* as important exposure determinants or confounders. *A priori* predictors included: age; years working in noisy environments; years working in scrap; years living at Agbogbloshie; overall averages for levels of noise exposure; maximums for noise exposures; averages for occupational noise exposures; levels of toxic elements Pb, Hg, Cd, As, and Mn (adjusted for interquartile range [IQR]); levels of essential elements Cu, Fe, Se, and Zn (adjusted for IQR); level of income; and number of times eating meat in a week (as an indicator of nutritional status). Occupational task-based analyses of hearing and noise exposure were conducted to determine the extent to which certain tasks may influence hearing over others. These included workers’ primary tasks (limited to one of four: dealing, dismantling, collecting, or other), as well as recently performed tasks (including all possible tasks reported on the survey: Pb battery recycling, smelting, repair, collection, sorting, removing wires, dismantling, burning, and sorting through ash).

Final models were identified using forward stepwise regression techniques with variables which showed significance or borderline significance in univariate models and then subsequently adding more *a priori* variables to improve the significance of the terms. Three final models were selected for three hearing outcomes (frequency-specific thresholds at 4 and 6 kHz and NN_D_, using Equation (5)) based on model performance, as measured by the highest coefficient of determination, adjusted R^2^. Principle component and factor analysis approaches in modeling groups of elements and job tasks were also utilized in regression modeling.

## 3. Results

Data collection occurred over seven days in April 2014. A total of 58 e-waste workers were recruited, with the majority (n = 32) recruited over three consecutive days in the middle of the data collection period. At least 20 workers refused to participate in the study; refusals were primarily due to schedule conflicts and disruptions in the Agbogbloshie community due to the death of a prominent local leader shortly before data collection began. A few workers denied interest in participation due to a low-level recruitment fee in comparison to their wages from dismantling waste.

Table 1 shows basic demographic characteristics of the sample. Participants were generally young adults (mean age of 26), low income (38% had daily income < 10 GHS, or about $3 US based on the 31 December 2014 exchange rate of $1 to 3.22 GHS), Muslim (97%), and had worked in e-waste activities while living near Agbogbloshie for approximately 6 years on average. Most workers (78%) moved to Agbogbloshie from the Northern region of Ghana. The majority of participants were single (38%) or married and living with their spouse (38%). Most had no formal education (53%). The vast majority of workers recruited lived and slept at the site itself (71%). Both the workers and the site were transient with no permanent structures or formal industries. Participants reported an average of 6.2 ± 4.2 years working on scrap or e-waste and worked 10.6 ± 2.9 h/day on average. Given this temporary nature and lower social status of workers, health was an overall concern; surveys showed that 46 percent of the workers felt their health was poor or fair (data not shown).

### 3.1. Noise

**Figure 1:**
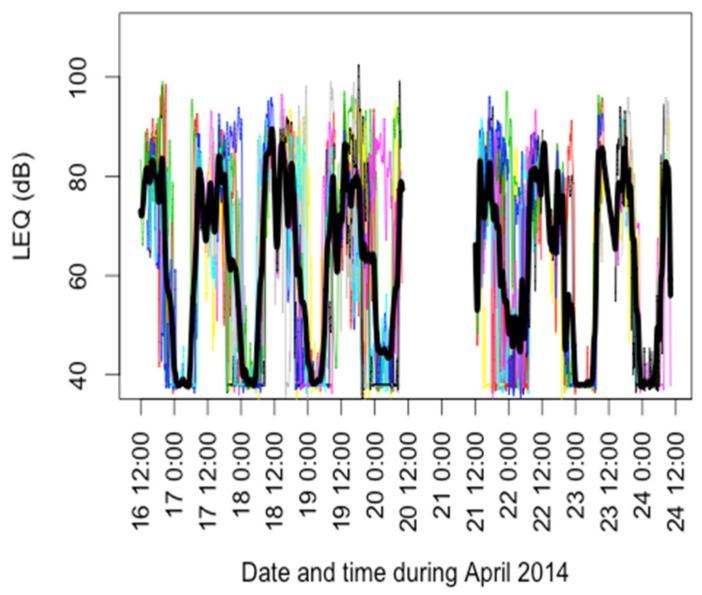
Individual noise exposures shown in different colors (N = 56) with splined average shown in black for e-waste recycling workers.

Participants had worked an average of 6.1 ± 4.2 years in loud noise (Table 1). For their current work in e-waste, the vast majority of participants (85%) reported being in loud noise very often (Table 2). Roughly half (464 h) of the 1090.6 h of noise data collected were below the dosimeters’ 70 dBA limit of detection (data not shown). L_EQ_ exposures measured over a large fraction of a day (19.1 ± 4.1 h on average) had a mean of 82.5 ± 3.8 dBA, with a range of 74.4–90.0 dBA (Table 2). The mean 8-h TWA was 84.6 dBA, just below the 85 dBA NIOSH recommended exposure limit, and 397 of the 3.75 min intervals (>24.8 h) exceeded 85 dBA. Splined individual noise exposures over the research period showed a diurnal trend in noise levels (Figure 1), with nighttime being the quietest time period, but many high (above the WHO recommended 70 dBA daily noise exposure limit) exposures were documented during daytime hours. The maximum noise level for each individual was 97.0 dBA on average, with a range of 87.5–110.0 dBA, indicating a potential for exposures to high levels of noise in e-waste work. Occupational periods showed a higher mean L_EQ_ (85.6 dBA compared to 81.6 dBA), though the L_max_ was higher on average during non-occupational times (96.0 dBA compared to 94.3 dBA). Seventy-nine percent of participants reported that exposures to loud noise were also common during non-occupational periods (data not shown). Noise exposures on site were dominated by impact sounds from hammers and chisels used to dismantle electric and electronic items. No workers reported, or were ever observed to be, wearing hearing protection devices. Researchers observed many workers actively engaging in social activities on the work site and in proximity to work-related activities including impact noise exposures. Muslims often practiced prayer and washing rituals a few feet away from noisy work activities.

### 3.2. Occupational Characteristics

Details of the nine e-waste activities reported by participants via survey are shown in Table 3. The vast majority of workers had participated in dismantling (86%) and sorting (84%) activities during the six months prior to the study, while relatively few workers had engaged in the more specialized activities of smelting (38%) and e-waste repair (35%). Nearly half of workers (48%) reported dismantling as their primary e-waste task for the past three months. Detailed reporting indicating occupational activities and durations was collected from only 16 (29%) of our participants; the remaining participants failed to complete and/or submit daily activity logs collected with dosimeters.

### 3.3. Blood Biomarkers

The mean level of blood Pb in participants, 97.2 ppb or 9.72 µg/dL (Table 4), was six times the US geometric mean of 15.2 ppb for adults over 20 [51]. The US Occupational Safety and Health Administration (OSHA) requires temporary medical removal from a job if a worker’s blood Pb level (BLL) is above 60 µg/dL (CFR § 1910.1025(k)(1)(i)(A)). None of our participants exceeded this level. However, the maximum BLL of 32.6 µg/L exceeded the Biological Exposure Index (BEI) of 30 µg/100 mL set by the American Conference of Governmental Industrial Hygienists (ACGIH) [52]. Average blood Cd in our sample was 2.98 ppb, eight times higher than the geometric mean of 0.378 ppb among US adults > 20 years of age [51]. The blood Cd levels among these workers varied greatly from 0.9 µg/L to 22.7 µg/L. Both OSHA and ACGIH set biological monitoring standards for Cd at 5 µg/L; the subject with the maximum blood Cd level exceeded these US standards.

Neither OSHA nor BEI limits are currently available for Mn or As. The average blood Mn level was 12.6 ± 4.2 µg/L (range 6.5–24.6 µg/L), and the blood Hg mean (1.8 ± 1.4 µg/L) and maximum (6.4 µg/L) levels among these workers were under 10 µg/L, well below the toxic threshold of over 50 µg/L [53]. Levels of measured essential elements Cu, Fe, Se, and Zn were 1.1 ± 0.7 mg/L, 440.1 ± 628.2 mg/L, 163.6 ± 48.5 µg/L, and 5.5 ± 1.9 mg/L, respectively. Blood elements and worker tasks were explored separately [44].

### 3.4. Hearing Outcomes

**Figure 2:**
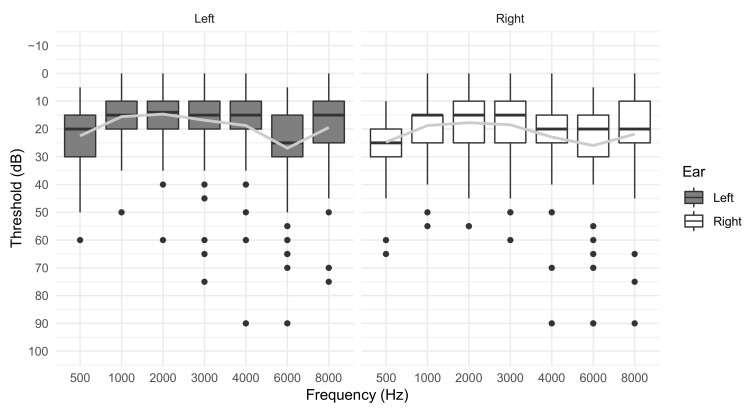
Audiometric hearing thresholds for e-waste recycling workers (N = 57).

Fifteen participants (25.9%) reported having trouble with their hearing, and five (8.9%) reported having had trouble since childhood or adolescence (data not shown). The majority of participants reported ringing in their ears after exposures to loud noise (67%), being bothered by occupational noise (76%), and being bothered by nighttime non-occupational noise (70%, data not shown). HTLs were worse in participants’ right ears for all frequencies except 6 kHz (Figure 2; descriptive statistics by frequency can be found in Table A1). Mean HTLs in both ears were <20 dB at all frequencies except 4 kHz and 6 kHz. Excluding low frequency hearing at 500 Hz (which may have been artificially increased due to background noise levels), HTLs were worst at 6 kHz and then 4 kHz. The pattern of mean HTLs across frequencies was consistent with a NN or noise-induced hearing damage (Figure 2), with damage worst at 4–6 kHz and recovery at 3 and 8 kHz. Variability in HTLs was greatest at 4 and 6 kHz, but quite high across all test frequencies, as shown by large standard deviation bars.

### 3.5. Noise Notch Analysis

More than half of participants were found to have a NN (Table 5). Of those having notches, the majority were bilateral. In both the right and left ears, notches were most frequently present at 6 kHz, followed by 4 kHz and then 3 kHz. When the four notch indices were compared, NN_D_ (which focused on 6 kHz) was found to have an average notch depth that was nearly twice that of the other indices. NN_D_ and left thresholds at 6 kHz were found to be significantly and negatively correlated (r = −0.66, *p* < 0.001). This fact, when combined with the high correlations between indices NN_A_ through NN_C_, and the majority of notches measured at 6 kHz, led to an exclusive focus on left-ear 6 kHz audiometric frequency and NN_D_ for subsequent analyses. The left ear was chosen and a conservative analysis was performed due to possible learning effects in the first ear (the right ear) tested in all participants; not one individual had previously ever had an audiometric test.

### 3.6. Bivariate Associations between Noise and Biomarkers Impacts on HL

Blood Pb, blood Cd, and blood Hg were not found to have meaningful bivariate relationships with HTLs or notch indices. Perceived noise exposure showed no relation to any of the HL measures. Negligible correlations were found between daily L_EQ_ and left ear HTLs at 6 kHz or NN_D_ (Spearman’s rho = 0.09, *p* = 0.51; −0.05, *p* = 0.71; respectively). L_max_ correlations with left HTLs at 6 kHz and NN_D_ showed similar non-significant results. Cu was somewhat linearly associated with L_EQ_ (data not shown). Blood Cu was also correlated with blood Cd, Pb, and Se (Spearman’s rho = 0.51, *p* = 0.0001; 0.58, *p* = 0.00001; and 0.40, *p* = 0.0003; respectively).

Zn was the only significant predictor of NN_D_, and for NN_A_ only Mn (*p* = 0.06) reached even borderline significance. Thresholds at 6 kHz were significantly predicted by Se, years working in scrap, and years working at Agbogbloshie. Since years working in scrap and years working at Agbogbloshie were highly colinear, only years working at Agbogbloshie was considered in further analysis.

### 3.7. Multivariate Regression Analyses

The results of our multivariate analyses for three hearing outcomes, both 4 and 6 kHz audiometric test frequency (Model 1 and 2, respectively) and NN_D_ (Model 3), are shown in Table 6. We started with the significant elements found for each outcome above in bivariate analyses and then forced several variables into both models based on our *a priori* assumptions: years living near Agbogbloshie, smoking status, number of times meat was eaten in a week, work diversity, and noise exposure. Age, a known risk factor for HL, was not forced into the model due to the young age of our sample population. Rather, we relied on the variable years lived in Agbogbloshie to account for duration of probable exposure to noise and elements. Biomarkers for toxicants, levels of essential elements, noise levels, and work tasks were added to the models sequentially to explore their impacts. Final models were selected based on adjusted R^2^, which ranged from 0.24 (Model 1) to 0.31 (Model 3).

For the 4 kHz model (Model 1), all included coefficients (Cd, Mn, number of times meat is eaten in a week, and years of life at Agbogbloshie) reached statistical significance. Increased levels of Mn, amount of meat eaten in a week, and length of time living at Agbogbloshie were associated with small but significant decrements in hearing ability, in the order of 6 dB HL per µg/L of these biomarkers in blood. Cd was unexpectedly associated with a slight improvement in hearing at 4 kHz. In the 6 kHz model (Model 2), a greater number of years living near Agbogbloshie, Se, and work diversity were each associated with significant decrements in hearing. Work diversity, age, Zn, Se, highest L_max_, daily L_EQ_, and age were all significantly associated with worse NN in the NN_D_ model (Model 3), while As was unexpectedly protective. High As levels are found in diets rich with seafood [54], so it is possible that higher As indicates better nutritional status.

Interactions between toxicant biomarkers and essential elements were explored. Significant interactions for NN_D_ included Pb*Zn (*p* = 0.02), Cd*Zn (*p* = 0.01), and Cu*Zn (*p* = 0.01). Among predictors of thresholds at 6 kHz, only Pb*Mn (*p* = 0.03) showed significance. Significant interactions as predictors of thresholds at 4 kHz were Mn*Zn (*p* = 0.03), Cu*Se (*p* = 0.03), Fe*Zn (*p* = 0.05), As*Zn (*p* = 0.04), and Fe*Se (*p* = 0.02).

One interaction (Zn and noise levels) improved the adjusted R^2^ of Model 1 and 2. There was a highly significant and consistent interaction between Zn and noise levels. This was across all hearing outcome metrics and multiple noise metrics. Thus, an interaction term for L_max_*Zn was included in the final models (Table 6). Se, Fe, and As showed significant interactions with certain noise metrics as well, however, these were not consistent (data not shown). Interactions between noise levels and Pb were borderline significant at 6 kHz thresholds for L_max_ (*p* = 0.07) and NN_D_ for maximum occupational exposures (*p* = 0.06). These interactions became significant only for thresholds at 6 kHz for average non-occupational noise (*p* = 0.04) and maximum non-occupational noise (*p* = 0.02). Due to the number of interactions explored, these *p*-values no longer showed statistical significance when multiple comparisons were taken into consideration.

## 4. Discussion

Given that threshold shifts in this population are likely to be permanent and hearing generally declines with age, we discovered high levels of HL and high noise in this population of young adult e-waste recycling workers. The oldest worker in our study was 60, so if it is assumed that these exposures are not unusual and might continue in workers from age 20 and continue for an additional 40 years, then hearing health is at serious risk. This is especially concerning due to the lack of hearing protection use and access to personal protective equipment for this population.

Our study indicated that these workers had unexpectedly high noise exposures during both occupational and non-occupational periods. These elevated noise exposures could help explain the decrements in HTLs observed among workers through audiometric testing—average right ear thresholds at 4 and 6 kHz were >20 dB, which, in a group of workers as young as those assessed (mean age just under 26 years), is much worse than would be expected in a similar group of noise-free workers [55]. By comparison, recent estimates among the subset of workers age 26–35 in the US workforce found that only roughly 3% of workers had mild or greater hearing loss with hearing thresholds of 25 dB or greater [56]. The high noise levels observed—the average occupational noise exposure was 85.6 dBA—combined with indications of NIHL (mean left NN depth of 9 dB HL) in many workers suggest that HL prevention efforts are warranted among this group. Additionally, subject report and researcher observations indicated that these workers never use hearing protection devices, though use of other protective equipment (e.g., boots, gloves, and thick aprons for activities with Pb-acid batteries) was reported and observed.

At Agbogbloshie, e-waste recycling work is conducted outdoors, and the workspace for e-waste recycling workers is often communal, with many non-work-related activities integrated spatially and temporally with work. This reinforces the notion that exposure assessment for these workers cannot simply focus on traditional occupational activities. Our noise exposure assessment on these workers, which spanned a period of up to 24 h, suggests that a holistic approach that integrates exposures from occupational and non-occupational activities is critical to understand the risk of NIHL among these workers, as exposures during non-occupational periods were similar in magnitude to, and in some cases exceeded, occupational exposures.

In addition to noise exposure and HL, we have documented exposures to toxic elements, along with blood levels for several essential elements, among the e-waste workers. Average levels of blood Pb in participants (mean = 9.72 µg/dL Pb) were eight times the US adult average. The average levels of blood Pb in participants were below the OSHA criterion for occupational medical removal at 40µg/dL. However, two participants (3%) had blood Pb levels higher than the more protective ACGIH BEI (30 µg/dL). Average blood Cd was over seven times higher in our sample than the average in US nonsmoking adults (mean = 2.98 ppb Cd). The highest blood Cd levels measured in participants were four times greater than that OSHA criterion of 5 µg/L.

The average blood Mn level in our participants (12.6 µg/L, SD = 4.2) was higher than the reported average manganese concentration in blood in healthy adults in the US (9 µg/L, range from 4–15 µg/L) [57]. South African manganese smelter workers with a mean blood manganese level of 12.5 µg/L (SD 5.6) showed significantly worse performance in neurobehavioral tests than workers with lower exposures [58]. Blood Mn alone does not constitute an adequate indicator of overexposure to Mn. Nevertheless, our participants may be at risk of health effects as a result of high blood Mn levels.

The average concentration of blood Se in our participants was 163.6 µg/L (range 72.6–319.3 µg/L), which is higher than the average level for all ages and both sexes of US population (124.8 µg/L) [59]. This level is also comparable to the average levels observed in other populations in Canada, China, Greece, Italy, Finland, New Zealand, and Germany, ranging from 59 µg/L to 206 µg/L (SD = 6~37) [60,61,62,63,64,65,66,67,68,69]. However, the blood Se concentrations in our participants had higher maximum and lower minimum levels [64,65,66,70] than those observed in other populations, which may reflect dietary variability or insecurity. Both Se overexposure and deficiency are associated with adverse health effects [71,72].

Arsenic (As) is cleared rapidly from blood, and As blood levels therefore indicate only very recent exposures. The average blood arsenic level in our participants was higher than typical values observed in non-exposed individuals (<1 µg/L) [73,74,75] indicating that dietary or drinking water exposures are likely. High levels of As in water and food have been observed in Ghana in the past [76,77,78]. Conversely, their blood Hg levels were within a normal range of healthy adults (<10 µg/L, toxic threshold >50 µg/L). [53].

The average levels for essential metals Cu, Zn, and Fe were similar to the reported average levels in non-exposed individuals [79,80,81]. However, individuals with higher levels of these metals may be at risk of some health effects [79]. Iron deficiency has been linked with HL in adults [82]; however, the effect of high levels of iron on hearing outcomes is not established.

Our finding of a significant interaction related to noise exposures and blood Zn levels has not previously been shown in the literature. Zn levels in blood are related to immediate dietary intake [83,84], and deficits have been shown to be associated with hearing problems [85]. Multivariate models using IQR-adjusted and centered data in this study showed that higher levels of Zn were protective when predicting auditory thresholds at 4 kHz and 6 kHz. However, the interaction between Zn and noise was positive as well as significant indicating that Zn may be potentially protective against noise, but at higher noise levels Zn is no longer able to protect against auditory threshold decline. In models predicting the NN depth, increasing levels of Zn were associated with deeper NN, or worse hearing, and the interaction term was no longer significant. While findings were somewhat contradictory, the number of models showing significance for Zn and maximum noise exposures suggest that this relationship warrants further explorations in future study.

Zn is an antioxidant and has been found to be protective against Cd ototoxicity in rats [86], and while not always consistent in this study, higher levels of zinc did show some capacity in our models for protection. This cohort demonstrated a large range of Cd exposure levels, compared to Hg for example, and this may play a role in the inconsistencies of our models. Se, a second antioxidant, showed significant protective effects in Model 3, both with and without the interaction term. However, Se was more inconsistent in protective direction across models. Selenium has been shown effective in treating idiopathic sudden sensorineural HL in people [87] and deserves further exploration in its roles involved with NIHL.

Given the paucity of studies on this vulnerable occupational group, it is difficult to compare our findings to those of other researchers. Previous literature has examined surface dust samples and determined extremely high hazard quotients at the Agbogbloshie waste recycling site for Pb and Cu, with levels of great concern to child health [46]. Cd and Zn also displayed concerning hazard quotients while Fe, Mn, chromium, and nickel were not of concern [46]. Our study found high blood values of Pb, Cd, and Cu in some participating adults supporting this study’s assertion of high levels of health risks due to Pb and Cd, especially concerning where children may be present on site. High levels of Zn and Cu were found in our study participants as well, highlighting the need to further explore these metals as contaminant concerns.

Some recent literature has explored metals and metalloids in the blood of e-waste workers [88,89,90]. One study examined levels of numerous trace elements in their urine and this study found high levels of As, which was not found to be due to drinking water, but likely fish and shellfish sources [42]. Cd was not found to be elevated in worker urine, however Fe, antimony, and Pb were after adjusting for age [42]. While our study did not examine antimony, we also found evidence to support concerning elevated Fe and Pb in blood samples. Asante et al. (2012) did not examine how the levels found may influence health outcomes of workers as we have done [42].

### Limitations

Our study had a number of limitations. The first and primary limitation was the small sample size, which may limit the range of interpretation and generalizability of our results. However, given the relatively homogeneity observed among e-waste recycling activities across Agbogbloshie, and the fact that this site is one of, if not the largest e-waste recycling sites in the world, even in the absence of generalizability our study results still have great utility. A second limitation pertains to the audiometric test results that form the primary outcome of the study. Conditions at the Ghana Post Clinic were not optimal for audiometric testing, which ideally would have been conducted in a sound-dampened audiometric test booth. As a result, it is possible that some of the hearing thresholds measured among our workers were artificially elevated by masking due to background noise, particularly in the low frequencies (e.g., <1000 Hz). Our use of insert-type earplugs and attenuating audiometric earcups reduced background noise at participants’ ears, but we cannot be certain that the attenuation achieved was sufficient to overcome masking effects. A third limitation resulted from insufficient detail collected from a number of workers at the completion of their noise dosimetry. This limited our ability to conduct task-specific noise exposure assessment, which might have provided useful information regarding activities that generated the highest noise levels. Our survey design, along with the delivery of the survey to the participants, represents a fourth limitation. We relied on self-reported information by participants, and given their strict religious expectations, there may have been some misreporting resulting in misclassifications. Translation provided by the translators hired for this study may have been inconsistent, resulting in misclassification and errors in participants’ survey results. In addition, some survey items were consistently omitted by one translator, reducing the amount of valid data obtained from participants he interviewed.

## 5. Conclusions

Our study has demonstrated that e-waste recycling workers at Agbogbloshie face substantial occupational health issues, including high exposures to noise and concerning levels of toxic metal biomarkers. These exposures appear to be associated with an increased risk of HL among these workers and indicate that HL prevention and metal exposure reduction efforts are needed among these workers as part of an overall effort to decrease occupational hazards and improve health outcomes among e-waste recycling workers. The relationship between metals and HL is still unclear and further study is needed. Further occupational hazard assessments are warranted among communities of e-waste workers in low-income countries such as Ghana, as their health is likely disproportionately impacted due to occupational hazards. Furthermore, exposures among these workers may grow worse over time with the expected increase in e-waste generation.

While the lack of formal employment arrangements eliminates the potential for an organized, employer-sponsored HL prevention program (HLPP), it is still possible for governmental or non-governmental organizations (NGOs) to provide support to protect workers’ hearing. The most basic and evident need for workers is training on occupational health hazards, including noise and metals. Hearing protection devices could also be distributed, though it should be noted that the likelihood of sustainable provision of protectors to the workforce is extremely small due to economic and logistic challenges and that such an effort would be largely useless in the absence of concurrent training. Expert guidance on methods for e-waste recycling that create the least noise possible could also be beneficial; this approach may have the added benefit of reducing exposures to metals, dust, and other occupational hazards.

## Figures and Tables

**Table 1:** Demographic information (N = 58 e-waste recycling workers).

**Variable**	Mean	SD
Age (years)	25.9	7.9
Estimated age (16/58 = 28%)	23.1	4.8
BMI (kg/m^2^)	22.8	2.3
Years living near Agbogbloshie	6.0	4.2
Years worked on e-waste *	6.2	4.2
Years worked in loud noise	6.1	4.2
Typical hours worked per day *	10.7	2.9
**Variable/Category**	**n**	**%**
Estimated (rather than known) age	16	28
Self-reported hearing difficulties	15	26
Sleep at Agbogbloshie	41	71
Muslim	56	97
Daily income (US dollar estimation ^)		
≤GHS 10 (~US $3)	22	38
GHS 11–20 (~US $3–6)	18	31
GHS 21–30 (~US $6–9)	7	12
GHS 31–40 (~US $9–12)	4	7
>GHS 40 (~US $12)	7	12
Marital status		
Separated or divorced	3	5
Married, living with spouse	22	38
Married, not living together	11	19
Single	22	38
Highest level of education		
None	31	53
Primary school	11	19
Middle school	12	21
Secondary school	4	7
Moved to Agbogbloshie from **		
Northern Ghana	42	78
Southern Ghana	9	17
Other location	2	4

* N = 55, ** N = 54, ^ exchange rate based on 31 December 2014 US $1 = 3.22 GHS.

**Table 2:** Daily noise measurement data (N = 56 * e-waste recycling workers).

Period/Variable	n	Mean	SD		Minimum		Maximum	
Overall								
Duration (h)	56	19.1	4.87		4.1		23.5	
L_EQ_ (dBA)	56	82.5	3.84		74.4		90.0	
L_max_ (dBA)	56	97.0	4.15		87.5		110.0	
Occupational period								
Duration (h)	17	1.3	2.61		0.5		13.4	
L_EQ_ (dBA)	17	85.6	3.52		78.6		90.2	
L_max_ (dBA)	17	94.3	4.53		85.7		102.5	
Non-occupational period								
Duration (h)	56	18.6	5.98		4.1		23.5	
L_EQ_ (dBA)	56	81.6	4.35		70.3		90.0	
L_max_ (dBA)	56	96.0	4.98		80.0		110.0	
	**Very Often**		**Fairly Often**		**Sometimes**		**Never & Almost Never**	
	**n**	**%**	**n**	**%**	**n**	**%**	**n**	**%**
Self-reported frequency of noise exposure at work (N = 58)	49	84.5	1	1.7	6	10.3	2	3.4

* 2 of 58 total participants did not complete a noise dosimetry measurement.

**Table 3:** Occupational characteristics reported via survey (N = 58 e-waste recycling workers).

			Yes	
Variable/Category		N	n	%
Activities performed in past six months *				
	Ash	57	32	56
	Burn	55	40	73
	Collect	55	41	75
	Dismantle	57	49	86
	Lead Batteries	57	27	47
	Sort	57	48	84
	Smelt	56	21	38
	Repair	57	20	35
	Remove wire (not by burning)	57	43	75
Primary e-waste activity (N = 58)				
	Collecting		10	17
	Dealing		12	21
	Dismantling		28	48
	Other		8	14

* N = 57; one participant did not respond to these questions.

**Table 4:** Element levels (ppb or μg/L) in whole blood (N = 58 e-waste recycling workers).

Element	Mean	SD	IQR	Minimum	Median	Maximum
Toxic Elements						
Arsenic (As)	4.63	2.73	4.05	0.75	3.76	12.08
Cadmium (Cd)	2.97	3.03	1.11	0.92	2.41	22.74
Lead (Pb)	97.2	57.8	59.2	29.9	81.8	326.1
Manganese (Mn)	12.6	4.2	4.5	6.5	11.8	24.6
Mercury (Hg)	1.8	1.4	1.4	0.5	1.3	6.4
Essential Elements						
Copper (Cu)	1061	716	201	566	927	6164
Iron (Fe) *	440,161	62,824	66,710	253,078	443,015	573,369
Selenium (Se)	163.6	48.5	55.2	72.6	152.4	319.3
Zinc (Zn)	5539	1965	1193	2392	5281	16,255

* N = 57; one sample of Fe not reported by lab.

**Table 5:** Noise notch results for individual frequencies and notch indices (N = 55 e-waste recycling workers *).

Variable/Category	n	%
Presence of noise notch		
None	20	40
Both right and left ears	16	32
Right ear only	10	20
Left ear only	9	18
Right ear notch		
At 3 kHz	7	13
At 4 kHz	10	18
At 6 kHz	15	27
Left ear notch		
At 3 kHz	7	13
At 4 kHz	11	20
At 6 kHz	17	31
**Notch index**	**Mean (dB)**	**SD (dB)**
NN_A_	−3.3	9.9
NN_B_	−3.8	9.6
NN_C_	−4.9	9.1
NN_D_	−9	10.3

* Noise notch analysis was not run on 2 of the 57 participants with complete threshold data. One of the two eliminated participants was due to age, the other due to profound deafness on the right side.

**Table 6:** Regression models predicting hearing outcomes in e-waste recycling workers (N = 55).

	Model before Interaction Term					Model Including Interaction Term				
Model/Variable	Adj. R^2^	*p*-Value	Coef.	SE	*p*-Value	Adj. R^2^	*p*-Value	Coef.	SE	*p*-Value
Model 1: Outcome of 4 kHz hearing threshold level *	0.28	6 × 10^−4^				0.39	1 × 10^−4^			
Intercept			17.34	2.23	5 × 10^−10^			15.64	2.15	5 × 10^−9^
Cadmium (Cd) ^†^^			−3.79	1.66	0.03			−2.63	1.64	0.1
Manganese (Mn) ^†^^			6.15	2.49	0.02			4.85	2.46	0.05
Number of times eat meat ^ (per week)			0.93	0.30	0.003			0.75	0.29	0.01
Years living at Agbogbloshie ^ (years)			1.37	0.52	0.01			1.63	0.49	0.002
L_max_ ^ (dBA)			-	-	-			−0.64	0.62	0.3
Zinc (Zn) ^†^^			-	-	-			−2.80	1.70	0.1
Interaction term: L_max_*Zn ^†^^			-	-	-			0.87	0.26	0.001
Model 2: Outcome of 6 kHz hearing threshold level *	0.24	0.002				0.39	1 × 10^−4^			
Intercept			24.55	2.01	0.1			22.98	1.89	1 × 10^−15^
Copper (Cu) ^†^^			2.14	2.11	0.3			4.04	2.04	0.05
Selenium (Se) ^†^^			6.43	2.60	0.02			6.46	2.43	0.01
Years living at Agbogbloshie ^ (years)			1.14	0.47	0.02			1.49	0.44	0.002
Work activity diversity ^ (range from 0 to 1)			13.62	10.89	0.22			7.51	10.13	0.5
L_max_ ^ (dBA)			-	-	-			−0.71	0.56	0.2
Zn ^†^^			-	-	-			−2.91	1.64	0.08
Interaction term: L_max_*Zn ^†^^			-	-	-			0.90	0.24	6E-4
Model 3: Outcome of notch index NN_D_ **	0.17	0.03				0.21	0.02			
Intercept			24.97	2.12	3 × 10^−15^			24.14	2.10	1 × 10^−14^
Zn ^†^^			0.30	1.66	0.86			−1.61	1.90	0.4
Arsenic (As) ^†^^			−3.05	3.15	0.3			−1.26	3.20	0.7
Se ^†^^			7.73	2.55	0.004			7.83	2.48	0.003
L_max_ * (dBA)			1.09	0.92	0.2			−0.04	1.07	0.9
Overall L_EQ_ ^ (dBA)			−0.92	0.95	0.3			−0.10	1.02	0.9
Work activity diversity ^ (range from 0 to 1)			21.61	11.94	0.08			16.26	11.93	0.2
Age ^			0.65	0.34	0.06			0.62	0.33	0.07
Interaction term: L_max_*Zn ^†^^			-	-	-			0.55	0.29	0.06

* Positive coefficients indicate worse hearing; ** Negative coefficients indicate worse hearing; ^†^ IQR adjusted—As: 4.0 μg/L; Cd: 1.0 μg/L; Cu: 206 μg/L; Mn: 4.4 μg/L; Se: 55 μg/L; Zn: 982 μg/L; ^ Variables are mean-centered after adjusting for IQR.

## Data Availability

The authors will make the data described in this manuscript available to other researchers upon reasonable request.

## Appendix A

**Table A1:** Audiometric hearing thresholds (N = 57 * e-waste recycling workers).

	Hearing Threshold Level (dB HL)			
Ear/Frequency	Mean	SD	Minimum	Maximum
Right				
500	25	11	10	65
1000 **	19	10	0	55
2000	18	12	0	55
3000	19	13	0	60
4000	23	16	0	90
6000	26	20	5	90
8000	22	17	0	90
Left				
500	23	13	5	60
1000 **	16	9	0	50
2000	15	11	0	60
3000	17	15	0	75
4000	19	19	0	90
6000	27	18	5	90
8000	20	18	0	75
Worst threshold	40	18	20	90
Right side	35	17	15	90
Left side	34	18	10	90

* One participant did not fit the testing headphones, ** two measurements were taken, however, only the second reading is reported here.
